# Diagnosis of a subarachnoid hemorrhage with only mild symptoms using computed tomography in Japan

**DOI:** 10.1186/s12883-016-0726-9

**Published:** 2016-10-18

**Authors:** Syuichi Tetsuka, Eiji Matsumoto

**Affiliations:** 1 Department of Neurology, Hospital of International University of Health and Welfare, 537-3, Iguchi, Nasushiobara, Tochigi 329-2763 Japan; 2 Department of Neurosurgery, Hospital of International University of Health and Welfare, 537-3, Iguchi, Nasushiobara, Tochigi 329-2763 Japan

**Keywords:** Subarachnoid hemorrhage, Computed tomography, Diagnosis, Headache

## Abstract

**Background:**

Japan is currently an aging society, with a huge proportion of elderly citizens. Consequently, the incidence and severity of subarachnoid hemorrhage (SAH) is predicted to increase in the future. Computed tomography (CT) is very important in the initial diagnosis of SAH. The proportion of hospitals owning CT systems in Japan is around four times greater than the mean number of systems owned by hospitals in other countries belonging to the Organisation for Economic Co-operation and Development. Because CT is readily available in Japan, it follows that this technique, with its impressive diagnostic power, might be more in demand in Japan compared to other countries. However, misdiagnosis of SAH is a relatively common problem and is associated with increased mortality and morbidity, even in individuals who initially present in good condition.

**Case presentation:**

We describe a patient with subtle clinical and CT signs of SAH. A 39-year-old Japanese man visited our hospital with a 3-day history of mild headache, shoulder stiffness, and a feeling of dizziness. His physical examination was normal aside from mild neck stiffness. Although CT did not reveal obvious abnormalities, we noticed subtle signs of SAH on CT images, which have been observed in SAH patients with mild symptoms. Thus, we diagnosed our patient with SAH and provided appropriate treatment (aneurysm clipping). Following this, the patient progressed without development of the initial complications, and he was subsequently discharged from our hospital without sequela.

**Conclusion:**

Thus, physicians should be able to recognize subtle characteristics of CT imaging in case of SAH patients with low grade symptoms, as this can facilitate early diagnosis.

## Background

The incidence of stroke and subarachnoid hemorrhage (SAH) in Japan is approximately 200 and 20 per 100,000/year for all ages, respectively [1, 2]. However, Japan is currently an aging society, with a huge proportion of elderly citizens. Consequently, the incidence and severity of SAH is predicted to increase in the future. Although there is an increasing incidence of SAH with age, it is in young otherwise healthy patients that is critically important not to miss SAH - a treatable disease in a neurologically intact person if diagnosed before a major hemorrhage.

A typical patient with SAH experiences thunderclap (frequently described as being the worst headache of his or her life) that develops during a period of physical exertion. This is often accompanied by a transient loss of consciousness. Patients with these classical findings present little diagnostic difficulty. However, in the absence of such signs and symptoms, physicians often miss the diagnosis of SAH, as several studies have demonstrated [3, 4].

Misdiagnosis, defined as the failure to correctly recognize SAH at first contact between a patient and a medical professional, continues to occur and is associated with increased mortality and morbidity occurs when subsequent major hemorrhage occurs several days later. For example, Kowalski et al. reported that 12 % cases were misdiagnosed from a total of 482 SAH patients admitted to a tertiary university hospital [5]. SAH is caused by the rupture of an intracranial aneurysm, which leads to the extravasation of blood under high pressure into the subarachnoid space. SAH triggers a cascade of events that can result in severe disability or even death. Poor outcomes are common and are typically due to early brain injuries associated with initial hemorrhage or are secondary to delayed cerebral ischemia and cerebral infarction. In misdiagnosed patients, the initial complications continue to develop and result in worse outcomes more often than in those who were initially diagnosed correctly [5]. Computed tomography (CT) is a very important tool in the initial diagnosis of SAH. In this study, we present a case of which diagnosis is difficult and describe some important points of clinical practice relating to the use of CT in the diagnosis of SAH in order to avoid misdiagnosis.

## Case presentation

**Fig. 1:**
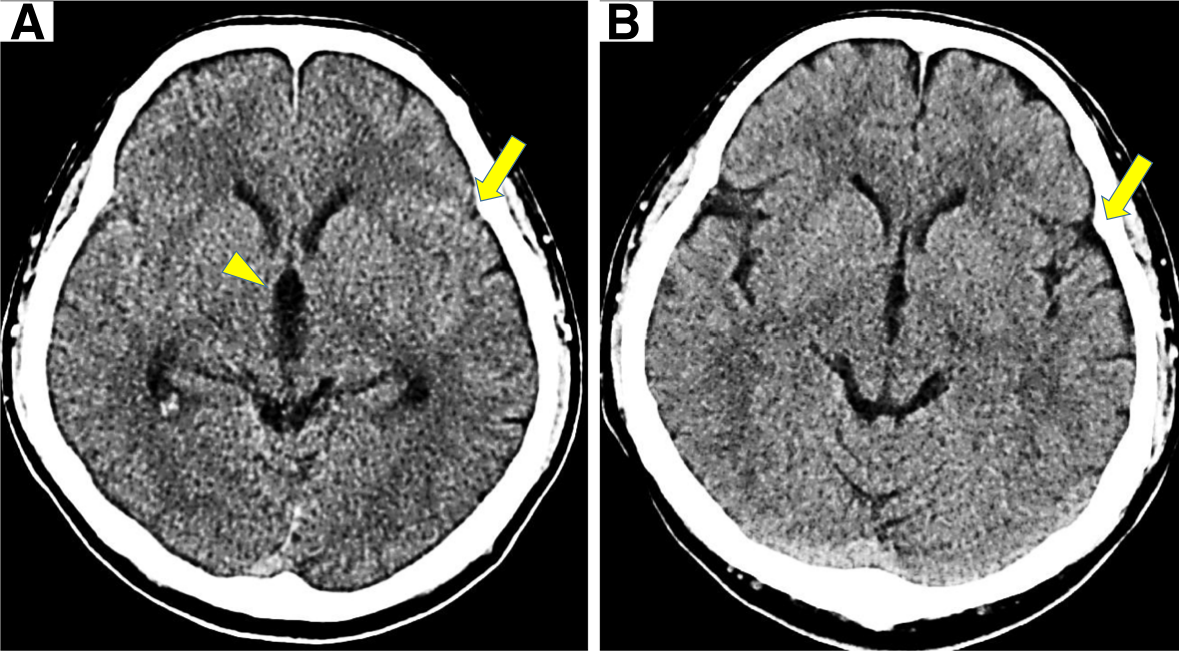
Computed tomography (CT) image of the current patient showing Sylvian fissures, which were not clearly visible bilaterally (**a**), compared to a CT image of the same patient taken 2 years ago when the patient complained of occipital headache, nausea, and vomiting. At that time, no abnormalities were detected (**b**). There was mainly isoattenuating SAH in the Sylvian fissures (slightly hyperattenuating on the left) and mild hydrocephalus - seen as enlargement of the third ventricle (**a**) in comparison to the previous CT (**b**). An *arrow* indicates Sylvian fissure, and an arrowhead indicates the dilated third ventricle

**Fig. 2:**
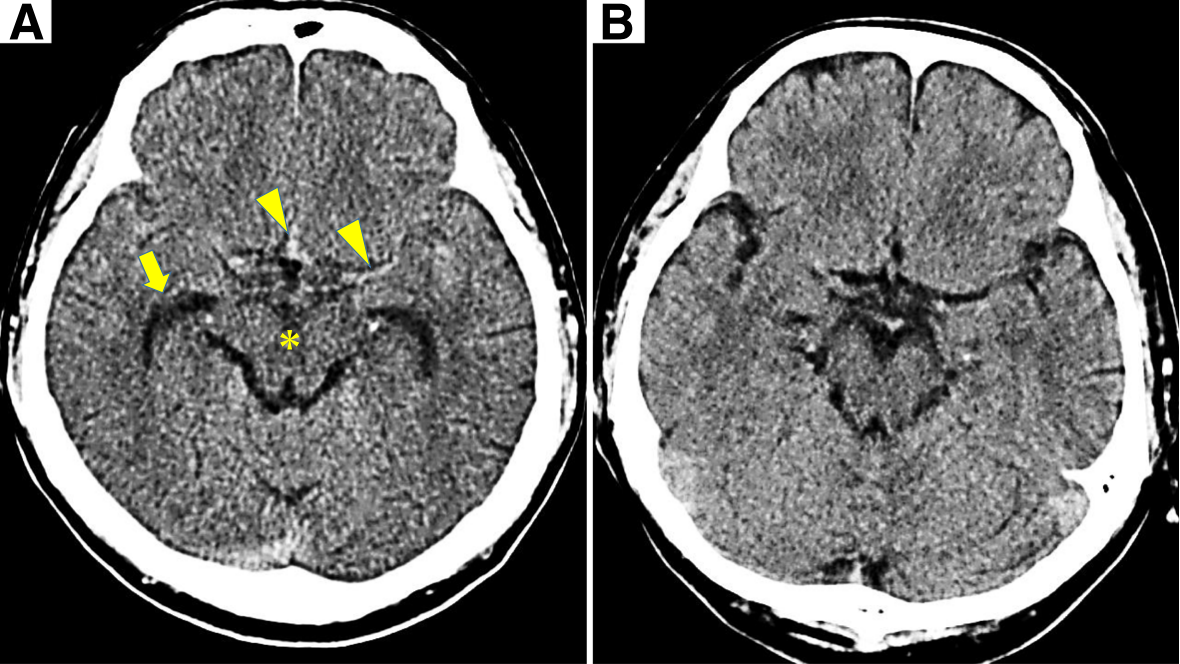
The slice is at level of the midbrain (mesencephalon) that traverses the infundibulum of third ventricle, interpeduncular cistern. In **a**, there appears to be hyperattenuating blood in the medial portion of the left Sylvian fissure and the anterior interhemispheric fissure, and shows mild dilation of the temporal horns of the lateral ventricles (looks like a moustache). In the center of the suprasellar cistern is CSF (hypoattenuating) in the dilated infundibulum of the third ventricle. These CT findings become clear in comparison to the previous CT (**b**). The SAH seen is in proximity to this left ACA aneurysm. An *arrow* indicates the temporal horns of the lateral ventricles. Arrowheads indicate hyperattenuating blood. An *asterisk* indicates CSF (hypoattenuating) in the dilated infundibulum of the third ventricle

**Fig. 3:**
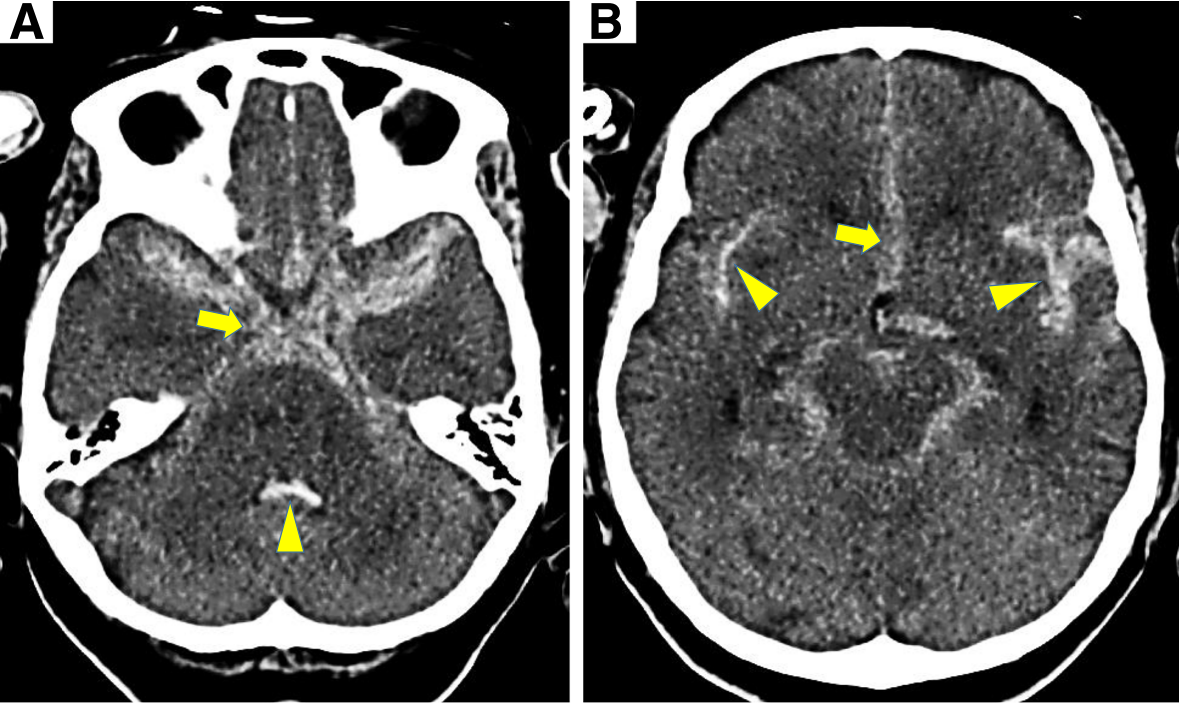
Typical non-contrast CT images of SAH showing blood in the subarachnoid space. The slice is at the level of the suprasellar cistern in **a**. Blood filling the basal cisterns, and there is hyperattenuating blood in the suprasellar cistern spreading laterally into the medial portion of the Sylvian fissures. In addition, there is blood that has refluxed into the fourth ventricle. An *arrow* indicates the blood in the suprasellar cistern. An *arrowhead* indicates blood in the fourth ventricle. The slice is at the level of the midbrain (mesencephalon) in **b**. There is blood in the perimesencephalic cistern extending anteriorly into the anterior interhemispheric fissure at the level of the midbrain. Blood extending bilaterally into the Sylvian fissures. An *arrow* indicates the blood in the anterior interhemispheric fissure and the blood in lateral extents of the Sylvian fissures are indicated by *arrowheads*

**Fig. 4:**
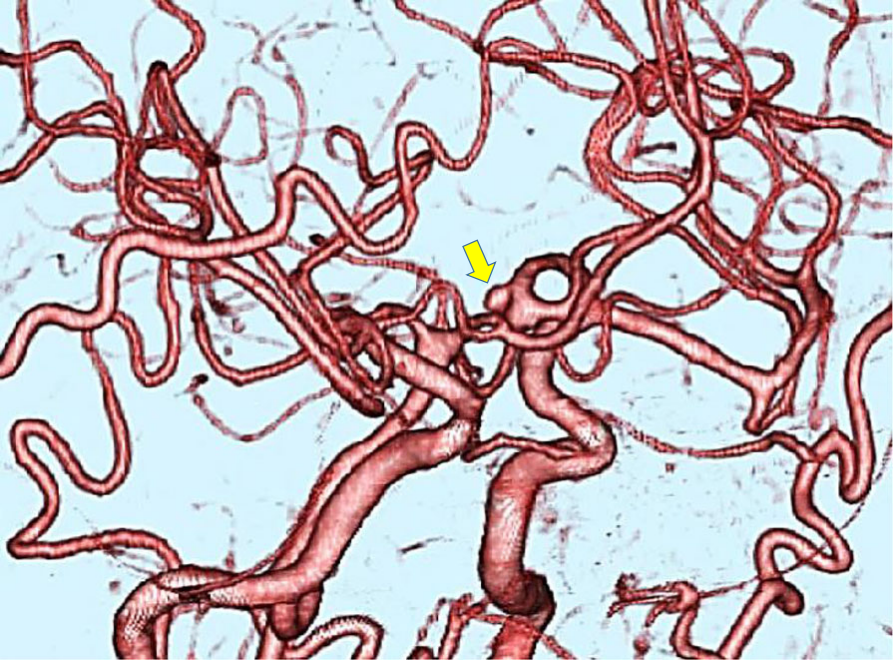
CT angiography (CTA) showing an aneurysm of which size was about 3.0 mm between A1 and A2 of the left anterior cerebral artery. The aneurysm is indicated by an *arrow*

A 39-year-old Japanese man visited our hospital with a 3-day history of mild headache, shoulder stiffness, and a feeling of dizziness. As the headache was only slight, he was able to remember what he was doing when the headache first developed. The patient was able to live a normal daily life prior to visiting our hospital with his symptoms. An initial examination on first presentation revealed that the patient was 170.0 cm in height, weighed 69.0 kg, and had a body mass index of 23.9. His blood pressure was 152/80 mmHg, with a regular pulse rate of 46 beats/min, and his body temperature was 35.9 °C. Physical examination revealed mild stiffness in the neck. No neurological findings were evident, and the patient’s laboratory data were normal. CT did not show any obvious abnormalities indicating hemorrhage or infraction. However, the Sylvian fissures were not clearly visible bilaterally due isoattenuating SAH in the Sylvian fissures (slightly hyperattenuating on the left) (Fig. 1a). Interestingly, the patient had visited our hospital 2 years ago complaining of occipital headache, nausea, and vomiting. At that time, CT was performed, which showed no abnormalities but provides useful normal comparison images (Fig. 1b). Another finding worth mentioning on Fig. 1a was mild hydrocephalus - seen as enlargement of the third ventricle - again, in comparison to the previous CT (Fig. 1b). Consequently, he was diagnosed with a tension headache. Comparing the current CT images to those from his previous visit, we observed a clear difference in the sulcus in the cerebral cortex, which appeared to be narrower and was accompanied by cerebral edema. In addition, Fig. 2a is at the level of the midbrain (mesencephalon) that traverses the infundibulum of third ventricle, interpeduncular cistern, there appears to be hyperattenuating blood in the medial portion of the left Sylvian fissure and extending into the anterior interhemispheric fissure, and shows mild dilation of the temporal horns of the lateral ventricles (looks like a moustache). In addition, the suprasellar cistern seem on this slice is filled with isoattenuating blood (evident in comparison with the normal Fig. 2b). In the center of the suprasellar cistern is cerebrospinal fluid (CSF) that is hypoattenuating in the dilated infundibulum of the third ventricle. These CT findings become clear in comparison to the previous CT (Fig. 2b). Thus, we suspected SAH, although the patient’s CT did not show obvious signs of SAH (Fig. 3). Next, CT angiography (CTA) revealed an aneurysm between A1 and A2 of the left anterior cerebral artery (Fig. 4). The patient received emergency neurosurgical clipping. He progressed after surgery without developing the initial complications and was discharged from our hospital without sequela.

## Discussion

### Clinical symptoms of patients with low grade SAH

One of the typical symptoms of SAH is the sudden onset of a severe headache, which has been described by 90 % patients who could give a history, as “the worst headache of my life” [6]. In most patients, the headache is accompanied by nausea and vomiting. The other physical findings in SAH patients are neck stiffness, diminished level of consciousness, papilledema, retinal and sub-hyaloid hemorrhage, third-nerve palsy, sixth-nerve palsy, bilateral weakness in the legs, abulia, nystagmus, ataxia, aphasia, hemiparesis, and left-sided visual neglect [7]. Such patients commonly complain of severe headache and are often taken in an ambulance to a local hospital. These symptoms were highly sensitive for identifying SAH [8]. Consequently, it is easy to rapidly diagnose SAH patients with moderate or severe symptoms using CT (Fig. 3). Figure 3a shows blood (hyperattenuating) through the suprasellar cistern and there also is blood in the anterior interhemispheric fissure and dilation of the infundibulum of the third ventricle. These findings would more strongly support the diagnosis of SAH in this patient. However, physicians often miss the diagnosis in low grade symptomatic SAH patients complaining of mild headache, neck pain, shoulder stiffness, and visual disturbance. It is therefore very important to pay careful attention to the care of these patients in order to prevent misdiagnosis. Moreover, these symptoms could not last long and diminish within a short time. Many of these patients are able to remember their situation when the symptoms first arise, as in the case of our patient.

### Consultation rate and medical care system for SAH patients with low grade symptoms

Because their symptoms are subtle, patients tend to present to the hospital much later. The current patient visited our hospital 3 days after developing headache. Such scenarios are often further complicated by the fact that these patients have high tolerance to pain and lead busy lives. Therefore, diagnosis using CT can become much more difficult. The incidence of SAH is known to increase with age, with a typical mean age of onset ≥ 50 years of age [9, 10]. Moreover, evidence of a gender-age effect on the incidence of SAH has emerged from pooled study data, with a higher incidence reported in younger men (25–45 years of age) [11]. Thus, physicians need to be particularly careful while examining younger men, like in the case of the current patient, as this group can be easily misdiagnosed. Because SAH patients with low grade symptoms often visit hospital after their work, physicians have a tendency to examine them outside normal outpatient hours. These are times when there may not be sufficient medical staff present. Consequently, medical examination through interview or laboratory examination, is likely to be insufficient. Therefore, there is high risk of misdiagnosis, defined as the failure to correctly recognize SAH at first contact between the patient and a medical professional. The headache experienced by these patients is sufficiently troublesome for them to purposefully visit a hospital outside normal hours. Thus, it is very important for physicians to use CT to identify the underlying cause. In addition, there are not many medical specialists in a local hospital during nighttime. We therefore consider that manual medical examinations are very important in this group of patients to prevent misdiagnosis.

### Diagnosis of symptomatic low grade SAH patients using CT

The first diagnostic study in these patients should be a non-contrast CT [12, 13]. The ownership ratio of CT systems in Japan is about 101 machines per 1,000,000 persons, referred to the Organisation for Economic Co-operation and Development (OECD) Health Statistics 2014. This ratio is about four times greater than the mean number of systems in countries belonging to OECD. Because CT systems are readily available in Japan, this technique may be more in demand, which can diagnose a range of medical conditions swiftly and accurately. The sensitivity of CT for SAH ranges from 90 to 95 %, implying that CT has a high sensitivity but should not be applied as a sole diagnostic modality for SAH diagnosis [14]. Moreover, CT sensitivity decreases with time since hemorrhage occurs. It has been shown that CT exhibits close to 100 % sensitivity within 12 h, 93 % within 1 day, and < 60 % within 1 week of hemorrhage [15]. The key CT finding of SAH is blood in the basilar cistern at the base of the brain where the Circle of Willis is located. The blood may then spread out from the basilar cisterns via the Sylvian fissures to the surface of the brain and into the subarachnoid spaces of the cortical sulci. Blood appears hyperattenuating (“hyperdense” = white on CT) in these regions where normally there is hypoattenuating (dark) CSF. If there is only a small quantity of blood which is mixed with CSF, it can appear isoattenuating (gray, similar to brain tissue) in these regions. This appearance is more subtle and can be difficult to identify with certainty. Small quantities of blood (hyperattenuating) are more reliably seen when CT is performed soon after headache onset - up to 6–12 h [16]. When there is only a small quantity of subarachnoid blood in the basilar cisterns, it dissipates over time. CT is therefore less sensitive at detecting SAH after 24 h. With symptomatic low grade SAH patients, bleeding occurs in small volumes and patients often visit a hospital after several days have passed, similar to the current patient. Consequently, CT does not show typical abnormalities of SAH in these patients, such as the basal cisterns filling with blood. However, if Sylvian fissures are not clearly visualized bilaterally, as in the case of our patient, and the difference between left and right visualization of Sylvian fissures are recognized, we should pay attention to minute details with suspicion of SAH. Subtle but important CT signs of hydrocephalus include dilation of the infundibulum of the third ventricle and dilation of the temporal (inferior) horns of the lateral ventricles (normally slit-like and barely visible in young adults). Dilation of the bodies and frontal horns of the lateral ventricles can be difficult to assess on CT when mild. A CT finding frequently associated with SAH is communicating hydrocephalus - enlargement of the ventricles, which can be mild; Ventricular dilation (hydrocephalus) is not always mild - but may be and is then a subtle secondary sign of SAH (a non-specific abnormality, but abnormal nonetheless). Communicating hydrocephalus is due to the presence of blood in the subarachnoid space which impedes CSF flow and results in dilation of the ventricles (all four ventricles). As people age, cerebral volume loss also can cause ventricular dilatation. Therefore, the finding of mild ventricular dilation is more significant in a younger adult - as in the case presented by the ours. The dilation of the lateral ventricles, and especially the temporal horns of the lateral ventricles are visually distinctive (“moustache sign”). The third ventricle can also be dilated including the infundibulum of the third ventricle in the middle of the suprasellar cistern. In addition, it is more important not to recognize cisternae that must be visible, and the sulcus in the cerebral cortex becomes narrower as a result of cerebral edema with symptomatic low grade SAH patients. In summary, when we perform a simple interpretation of CT in which white areas represent hemorrhage and black represents infraction, we are unable to detect SAH with low grade symptoms. Table 1 shows a summary of characteristics of CT imaging, which are important in the diagnosis of symptomatic low grade SAH patients. Because the sensitivity of CT decreases in SAH patients when several days have elapsed since the initial onset of symptoms, it is often difficult to clearly visualize important clinical features. SAH is absorbed by the arachnoid granulations of the parietal region via fissura sylvia. Thus, when SAH patients with low grade symptoms visit a hospital during sub-acute periods, hematoma may be observed only in the parietal region. However, even in such cases, it might not be possible to clearly observe hematoma. Patients with small hemorrhages, who are the most likely to receive an incorrect clinical diagnosis, are also more likely to have negative results or misinterpretation on CT [17, 18]. Although magnetic resonance technology is continually advancing and can detect aneurysms, standard magnetic resonance imaging (MRI) is inferior to CT in terms of detecting acute SAH [19]. Although MRI with fluid-attenuated inversion recovery shows promise [20], CT remains the imaging method of choice because of its wider availability, lower cost, and greater convenience for patients and also because there is wider experience in terms of its interpretation. In addition, the role of MRI in perimesencephalic SAH is controversial [21]. Another approach is the use of CT followed by CT angiography (CTA). The sensitivity and specificity of the CTA approach for detecting aneurysms > 3 mm are currently approaching 100 % [22, 23].

**Table 1:** Characteristics of CT imaging in SAH patients with low grade symptoms

Finding of blood that is hyperattenuating or, if slight and admixed with CSF, appears isoattenuating in the basilar cisterns
Sylvian fissures which are not clearly visualized bilaterally
Differences between left and right visualization of Sylvian fissures
Ability to recognize basal cisterns or cisternae
The sulcus in the cerebral cortex becomes narrower as a result of cerebral edema
Ventricular dilation (The lateral ventricles, and especially the temporal horns of the lateral ventricles are visually distinctive; “moustache sign” and the third ventricle can also be dilated.)
Blood refluxed into the fourth and sometimes third ventricle

### Prognosis of SAH cases initially misdiagnosed

In a previous study, the misdiagnosis of SAH occurred in 5.2–32 % patients and was associated with smaller hemorrhage and normal mental status [5, 7]. Among individuals who initially present in good condition, misdiagnosis is associated with increased mortality and morbidity. Although both low and severe grade symptoms of SAH are caused by the rupture of an intracranial aneurysm, the initial complications develop in patients with an incorrect diagnosis. This results in worse outcomes that are four times more common in such patients than in those initially diagnosed correctly [5]. Because the extent of SAH is smaller in patients with low grade symptoms, the development of cerebral vasospasm is rare. Thus, if these patients receive appropriate diagnosis and treatment, they will be able to make the transition back to civilian life, similar to the current patient.

## Conclusion

Because SAH triggers a cascade of events that could result in severe disability or death, it is crucial that appropriate diagnosis and treatment are provided in a timely manner. In particular, many SAH patients with low grade symptoms are often misdiagnosed. In those who initially present in good condition, misdiagnosis is associated with increased mortality and morbidity. Lowering the threshold for CT scanning in patients with mild symptoms that are suggestive of SAH may help reduce the frequency of misdiagnosis. Thus, physicians should be able to recognize the specific characteristics of CT imaging that are associated with SAH patients with low grade symptoms.

## Abbreviations

CT: Computed tomography
CTA: CT angiography
MRI: Magnetic resonance imaging
OECD: Organisation for Economic Co-operation and Development
SAH: Subarachnoid hemorrhage
